# Expression of Helper and Regulatory T Cells in Atopic Dermatitis: A Meta-Analysis

**DOI:** 10.3389/fped.2022.777992

**Published:** 2022-04-01

**Authors:** Dao-jun Zhang, Fei Hao, Tian Qian, Hai-xing Cheng

**Affiliations:** Department of Dermatology and Plastic Surgery, The Third Affiliated Hospital of Chongqing Medical University, Chongqing, China

**Keywords:** Th22 cells, Th17 cells, IL-17, tregs, atopic dermatitis

## Abstract

**Background:**

Atopic dermatitis (AD) is a common inflammatory skin disease, with the incidence peaks in infancy. A meta-analysis was performed to assess the levels of T helper type 22 (Th22) cells, T helper type 17 (Th17) cells, interleukin (IL)-17, and Tregs in peripheral blood of patients with AD.

**Methods:**

A comprehensive literature search was performed in PubMed, Embase, China National Knowledge Internet, and Wan-fang Data from the day of inception of this study to July 2021. Two authors independently extracted the data, which were pooled and calculated using Stata software version 15.

**Results:**

A total of eight studies met the inclusion criteria. Compared with control group, patients with AD had an increased proportion of Th22 cells [weighted mean difference (WMD) = 2.07, 95% CI (1.33, 2.81), *p* < 0.001], Th17 cells [WMD = 1.04, 95% CI [0.66, 1.43], *p* < 0.001], IL-17 [WMD = 17.56, 95% CI (11.1, 24.03), *p* < 0.001], and a decreased proportion of Tregs [WMD = −2.49, 95% CI (−2.93, −2.05), *p* < 0.001] in peripheral blood. The subgroup analysis showed that patients with higher disease severity had higher levels of Th22 [mild: WMD = 1.33, 95% CI (1.24, 1.41), *p* < 0.001; moderate: WMD = 1.41, 95% CI (1.36, 1.54), *p* < 0.001; severe: WMD = 3.46, 95% CI (3.34, 2.81), *p* < 0.001] and lower levels of Tregs [mild: WMD = −1.43, 95% CI (−1.75, −1.11), *p* < 0.001; moderate: WMD = −2.16, 95% CI (−2.46, −1.86), *p* < 0.001; severe: WMD = −2.96, 95% CI (−3.25, −2.67), *p* < 0.001] in peripheral blood compared to healthy controls.

**Conclusion:**

The random effect model of the meta-analysis showed patients with AD had an increased proportion of Th22 cells, Th17 cells, and IL-17, whereas a decreased proportion of Tregs was found in peripheral blood. The results demonstrated that Th22 cells, Th17 cells, IL-17, and Tregs may be involved in the pathogenic mechanisms of AD.

## Introduction

Atopic dermatitis (AD) is a common inflammatory skin disease, with an increasing prevalence worldwide, affecting 15–20% of people in developed countries (1, 2). It can manifest at any point in life, but the incidence peaks in infancy, with onset occurring before six years of age in an estimated 80% of patients. It is mainly characterized by skin barrier dysfunction and pruritus, leading to recurrent eczematous lesions and a negative impact on the quality of life of these patients (3–5). AD has a complex pathogenesis that involves epidermal hyperplasia, barrier dysfunction (increased trans-epidermal water loss and decreased lipids), and immune activation (6–9). AD is a more frequent T cell-mediated skin disease. T helper and regulatory T cells (Tregs) play a pivotal role in immune suppression and are integral to the control of allergic responses in AD (10–12).

Under lineage-specific culture conditions, T helper cells develop from a naïve cluster of differentiation 4^+^ (CD4^+^) T cells and are nominated by their lineage-specific cytokines (13). It has been reported that T helper type 2 (Th2) cells were classically considered to be the main pathogenic axis in AD (14). Currently, T helper types 22 and 17 (Th1 and Th22, respectively) cells have been considered to be involved in the development of AD (15). Tissue homing Th22 cells exhibit anti-inflammatory, antiviral, and antibacterial activities and play a critical role in epidermal wound healing, allergies, autoimmune diseases, intestinal diseases, and tumors (16). Th17 cells are one of these CD4^+^ T helper cell subsets and play a critical role in the development of autoimmune diseases such as psoriasis (17). Interleukin (IL)-17 is mainly produced by Th17 cells and infiltrates the papillary dermis of AD lesional skin. The percentage of IL-17-producing CD4^+^ T cells in peripheral blood from AD patients is increased and associated with the severity of AD (18).

Tregs are recognized as a critical etiological factor for AD. It is well accepted that Tregs are critical for maintaining peripheral tolerance, preventing autoimmune diseases, and limiting inflammatory responses (19). Tregs can prevent excessive immune response by inhibiting the activity and proliferation of Teff cells to enhance immune tolerance and maintain immune homeostasis (20).

Various studies indicate that Th22 cells, Th17 cells, IL-17, and Tregs play a critical role in the pathological process of AD. Recognizing that individual studies might not be able to provide sufficient data on their own, we systemically reviewed original studies and performed a meta-analysis to evaluate the levels of Th22 cells, Th17 cells, IL-17, and Tregs in inflammation and pathology of patients with AD.

## Materials and Methods

### Search Strategy

This meta-analysis was developed according to Preferred Reporting Items for Systematic Reviews and Meta-Analyses (PRISMA) guidelines (21). Searches were conducted in PubMed, Embase, China National Knowledge Internet, and Wan-fang Data for articles published in English and Chinese from the day of inception of this study to July 2021. We used the following keywords in searching the databases: (helper T cells OR Th17 OR Th22 OR regulator T cells or Tregs) AND (atopic dermatitis OR AD). Two researchers (FH and QT) independently screened and selected the studies. Gray literature was not included in our search and studies were limited to research in humans.

### Inclusion and Exclusion Criteria

Articles met the inclusion criteria if they satisfied the following conditions: (i) the study was designed as a case-control study; (ii) participants included both AD patients and healthy controls; (iii) studies assessing levels of at least one of the cells and related cytokines of Th22 (CD3^+^CD4^+^IL-17A^–^IFN-γ^–^IL-22^+^) cells, Th17 (CD3^+^CD8^–^IL-17^+^) cells, Tregs (CD4^+^CD25^+^), or IL-17 in peripheral blood; (iv) T cells were detected by flow cytometry, while cytokines were measured by enzyme-linked immunosorbent assay; (v) studied patients had no age restrictions; and (vi) the severity of disease was determined by the scoring atopic dermatitis (SCORAD).

Exclusion criteria were as follows: (i) duplicated literature; (ii) reviews, conference abstracts, and editorial comments; (iii) animal research, basic research literature, existing meta-analysis, and systematic evaluation; and (iv) the literature of research subjects combined other system diseases, immune-related diseases, and other skin diseases.

### Data Extraction and Study Quality Assessment

In further data processing, two researchers (FH and QT) independently extracted data from all eligible studies. Excel spreadsheets were used in building extracted data from included articles. The characteristics extracted from each study included the name of the first author, publication year, the sample size of the study groups (case group and control group), age of the patients, and the mean and SD of peripheral blood cytokines.

The Newcastle-Ottawa Scale (NOS) was applied to assess the quality of all studies. The NOS checklist contains three parameters of quality, which are as follows: (i) selected population, (ii) comparability of groups, and (iii) assessment of either the exposure or outcome of interest for case-control or cohort studies. Each study was assigned a score of 0–9. The studies that scored greater than or equal to 7 were considered to be high-quality articles (22).

### Data Analysis

Meta-analysis was conducted using the Stata software version 15. Pooled effect calculated by using evaluation weighted mean difference (WMD) or odds ratio (OR) with 95% CI. Heterogeneity between studies was assessed using the Cochran Q test and *I*^2^ (23). If there was low heterogeneity (*p* > 0.1, *I*^2^ < 50%), a fixed-effect model was used to calculate the pooled effect; otherwise, a random-effect model was used (*p* < 0.1, *I*^2^ ≥ 50%) (22). To assess the robustness of the results, we performed sensitivity analyses by sequentially removing each study and rerunning the analysis. Egger’s linear regression test was used to detect publication bias. All results in this analysis were considered significant with a *p* < 0.05.

## Results

### Characteristics of Included Studies

**FIGURE 1:**
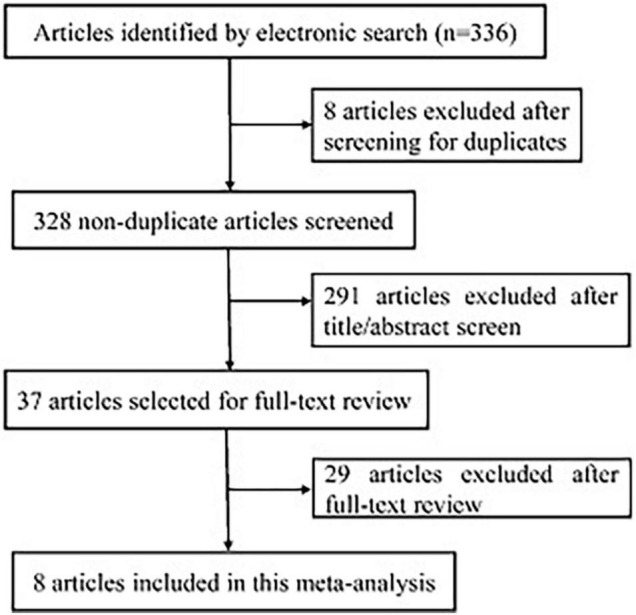
Flowchart of the study selection process.

The study selection process is shown in Figure 1. The initial literature search on the electronic databases yielded 336 potentially relevant records, which were reduced to 328 records after the removal of duplicates. Based on the preliminary screening of titles and abstracts, 291 records were excluded. The eligibility of the remaining 37 records was assessed by a detailed evaluation of the full-text articles. Following a full-text review, 29 articles were excluded due to incompatible with the inclusion criteria. Therefore, eight studies complied with the inclusion criteria and were included in this meta-analysis. The severity of AD was assessed by the SCORAD in these included studies. Interestingly, the population of the included studies was all Chinese adolescents. Study selection, quality of studies, and characteristics of included studies were described in Table 1.

**TABLE 1:** The characteristics of the studies included in this meta-analysis.

Authors (Year)	Group	Sample	Age	Th22	Treg	Th17	IL-17	Quality score
Li et al. (24)	Case	52	–	–	–	–	–	7
	Mild AD	20	–	2.11 ± 0.21	4.20 ± 0.44	–	–	
	Moderate AD	18	–	2.24 ± 0.23	3.64 ± 0.37	–	–	
	Severe AD	14	–	4.25 ± 0.30	2.81 ± 0.37	–	–	
	Control	20	–	0.77 ± 0.11	5.78 ± 1.2	–	–	
Su et al. (25)	Case	104	10.27 ± 2.41	–	–	2.25 ± 0.55	27.33 ± 4.22	7
	Control	35	10.82 ± 1.98	–	–	1.39 ± 0.27	15.63 ± 2.31	
Yu et al. (26)	Case	86	5.39 ± 4.04	–	–	1.3 ± 0.62	–	8
	Control	81	5.62 ± 2.02	–	–	0.63 ± 0.27	–	
Ren et al. (27)	Case	60	–	–	–	–	–	8
	Mild AD	22	–	2.11 ± 0.31	–	–	–	
	Moderate AD	20	–	2.23 ± 0.31	–	–	–	
	Severe AD	18	–	4.24 ± 0.38	–	–	–	
	Control	20	10.31 ± 2.02	0.81 ± 0.12			–	
Gao et al. (28)	Case	52	2-14	–	2.29 ± 0.67	1.2 ± 0.41	–	7
	Control	30	4-14	–	5.59 ± 0.45	0.54 ± 0.28	–	
Ma et al. (29)	Case	37	11.35 ± 3.01	–	1.99 ± 0.47	1.31 ± 0.39	–	6
	Control	33	11.64 ± 2.52	–	5.08 ± 1.18	0.41 ± 0.12	–	
Cui et al. (30)	Case	126	11.23 ± 4.68	–	3.44 ± 0.56	2.81 ± 0.63	30.84 ± 8.57	8
	Mild AD	50	–	–	4.31 ± 0.78	–	–	
	Moderate AD	42	–	–	3.50 ± 0.52	–	–	
	Severe AD	34	–	–	2.71 ± 0.31	–	–	
	Control	60	11.58 ± 4.94	–	5.67 ± 1.26	1.02 ± 0.21	11.25 ± 3.06	
Ma et al. (29)	Case	41	10.07 ± 3.45	–	2.01 ± 0.57	1.77 ± 0.55	33.24 ± 7.06	8
	Control	38	10.13 ± 3.31	–	5.04 ± 1.44	0.39 ± 0.15	11.68 ± 2.67	

*AD, atopic dermatitis.*

According to the NOS checklist, seven studies with scores ≥7 were considered high quality, while the remaining one was medium quality with a score of 6.

### Meta-Analysis

**FIGURE 2:**
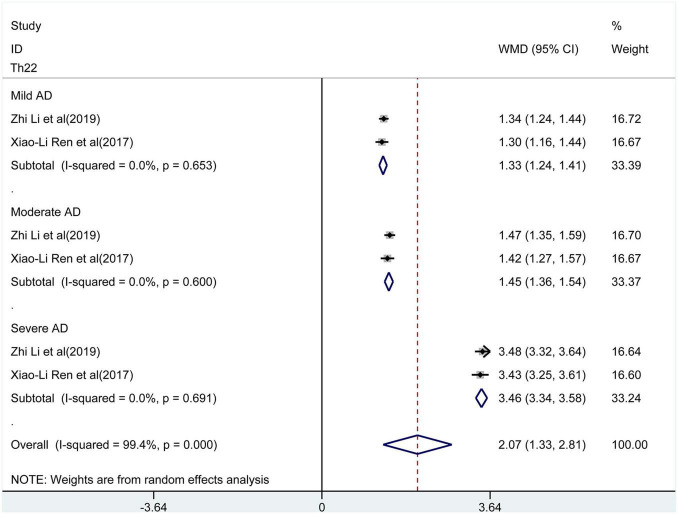
Forest plot of the levels of T helper type 22 (Th22) cells.

**FIGURE 3:**
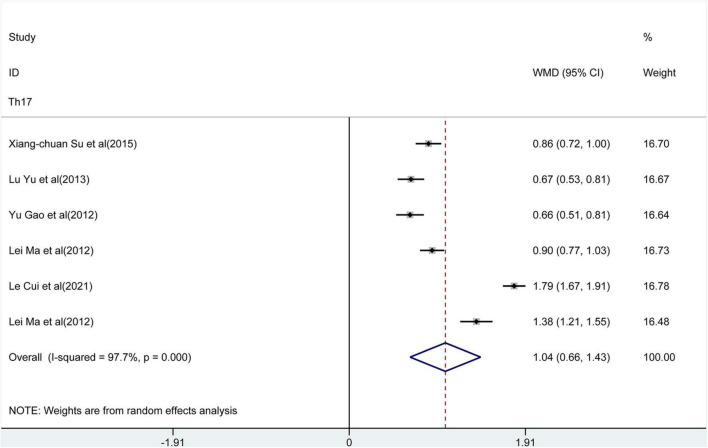
Forest plot of the levels of T helper type 17 (Th17) cells.

**FIGURE 4:**
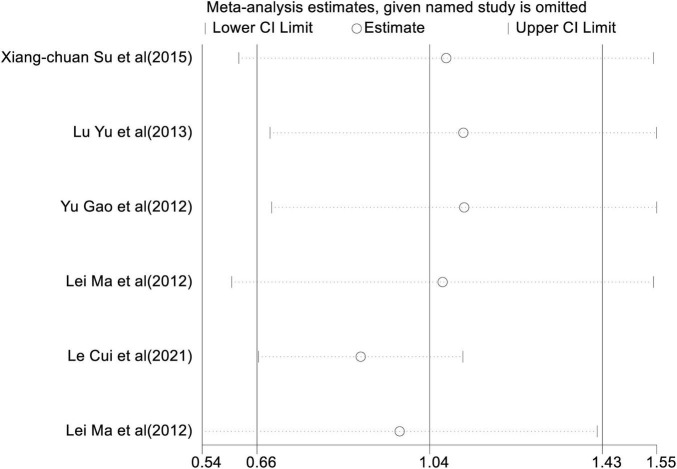
Sensitivity analysis of the levels of Th17.

**FIGURE 5:**
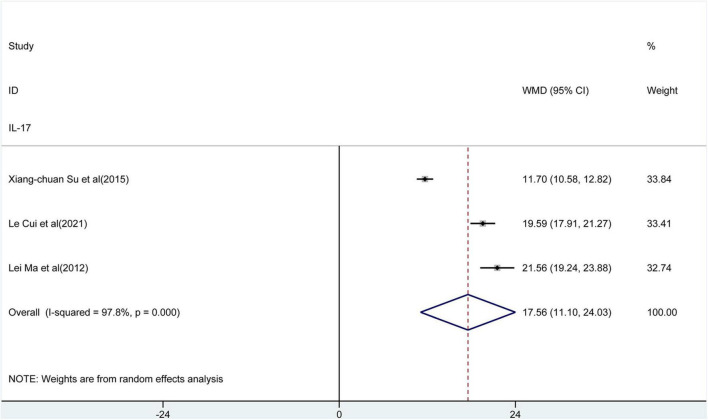
Forest plot of the levels of interleukin (IL)-17.

**FIGURE 6:**
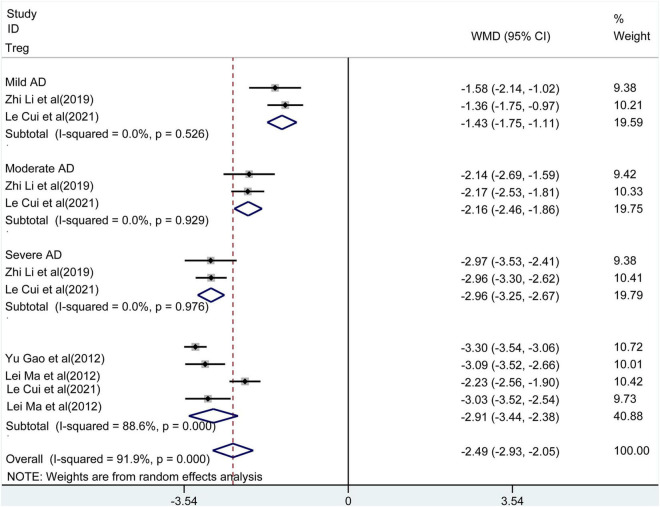
Forest plot of the levels of Treg.

The meta-analysis included the levels of Th22 cells, Th17 cells, IL-17, and Tregs in peripheral blood. The results of the meta-analysis are shown in Figures 2–6.

### The Percentage of Th22 in the Peripheral Blood of Patients With AD

Only two studies reported Th22 cells. Pooling the data of these studies showed (Figure 2) a significantly increased proportion of Th22 in peripheral blood compared to control groups (WMD = 2.07, 95% CI [1.33, 2.81], *p* < 0.001). Considerable heterogeneity existed between the studies (*I^2^* = 99.4%, *p* < 0.001). The subgroup analysis was performed based on the severity (mild, moderate, and severe) of the disease. The results showed that patients with higher disease severity had higher levels of Th22 compared to healthy controls [mild: WMD = 1.33, 95% CI (1.24, 1.41), *p* < 0.001; moderate: WMD = 1.41, 95% CI (1.36, 1.54), *p* < 0.001; severe: WMD = 3.46, 95% CI (3.34, 2.81), *p* < 0.001]. There was no heterogeneity detected in these results.

### The Percentage of Th17 in the Peripheral Blood of Patients With AD

In a pooled analysis of all 6 studies, the meta-analysis revealed (Figure 3) that there was a significant increase in the percentage of Th17 in peripheral blood of patients with AD compared to control groups [WMD = 1.04, 95% CI (0.66, 1.43), *p* < 0.001]. However, there was extensive heterogeneity among studies (*I*^2^ = 91.9%, *p* < 0.001). The results of the sensitivity analysis observed (Figure 4) that the direction of the pooled estimates was relatively robust [95% CI (0.66, 1.43)], which supported the main conclusions. There was no publication bias based on Egger’s test in this analysis.

### The Percentage of IL-17 in the Peripheral Blood of Patients With AD

Three studies evaluated the percentage of IL-17 in the peripheral blood of patients with AD. The results (Figure 5) indicated that the percentage of IL-17 in peripheral blood of patients with AD was significantly higher than control groups [WMD = 17.56, 95% CI (11.1, 24.03), *p* < 0.001]. Considerable heterogeneity existed among studies (*I^2^* = 97.8%, *p* < 0.001).

### The Percentage of Tregs in the Peripheral Blood of Patients With AD

Tregs had been reported in five studies. The meta-analysis observed (Figure 6) that the percentage of Tregs in peripheral blood of patients with AD was lower compared to controls [WMD = −2.49, 95% CI (−2.93, −2.05), *p* < 0.001]. High heterogeneity between studies was also detected (*I^2^* = 91.9%, *p* < 0.001). The subgroup analysis reflected lower percentage of Tregs in patients with higher disease severity compared to healthy controls [mild: WMD = −1.43, 95% CI (−1.75, −1.11), *p* < 0.001; moderate: WMD = −2.16, 95% CI (−2.46, −1.86), *p* < 0.001; severe: WMD = −2.96, 95% CI (−3.25, −2.67), *p* < 0.001]. No heterogeneity was observed among studies.

Therefore, the random-effect model of the meta-analysis was applied in preparing the four forest plots.

## Discussion

In this study, we presented a meta-analysis assessing the percentage of Th22, Th17, IL-17, and Tregs in the peripheral blood of patients with AD. The available data showed an increased proportion of Th22, Th17, and IL-17, whereas a decreased proportion of Tregs was found in peripheral blood compared to healthy controls.

Evidence showed that CD4^+^ T cells are one of the main cells in the immune system associated with autoimmune diseases. The naive CD4^+^ T cells can differentiate into several subclasses including Th1, Th2, Th3, Th9, Th17, and Th22, which produce different cytokines and chemokines to promote a specific type of immune response (24). It has been demonstrated that Th22 and Th17 cells immune responses contribute to chronic skin lesions of AD, especially in pediatric, intrinsic, and Asian patients (25). Interestingly, a total of eight articles were included in the present study, and the population was all Chinese adolescents. The results of this study will further confirm the above findings of the previous study (25). Lesional skin of AD involves elevated levels of the inflammatory cytokine Th22 and IL-22. Our results showed an increased proportion of Th22 in peripheral blood compared to healthy controls. Similarly, Nograles et al. also found that an elevation of production of Th22 was strongly increased in lesional skin of patients with AD (26). Th17 cells are essential in clearing pathogens during host defense reactions and can function as an up-modulator in skin lesions of AD (27). Koga et al. have demonstrated an elevation of the percentage of Th17 cells in peripheral blood of patients with AD (17), which is in line with findings from the present meta-analysis. Similarly, our findings are consistent with previously reported Th17 cells increased in skin specimens (28). IL-17 is mainly produced by Th-17 cells and infiltrated in the lesional skin of AD. In wounded skin, IL-17 plays important role in wound healing, tissue regeneration, and carcinogenesis (18). In our current study, the results were consistent with the previous studies, which demonstrated the percentage of IL-17-producing CD4^+^ T cells in both skin specimens (26, 28) and peripheral blood (17) of patients with AD was increased. These findings confirm the reliability of our meta-analysis, supporting the levels of Th22, Th17, and IL-17 involvement in the development and pathogenesis of AD.

Tregs play a critical role in maintaining self-tolerance and preventing autoimmunity, allergy, and inflammatory reactions. Evidence indicated that the percentage of Tregs was increased in the peripheral blood in the patients suffering from AD (29, 30). However, a previous study (28) revealed that Th17 cells and Tregs present mutually antagonistic function, and the percentage of Tregs negatively correlated with Th17 cells frequency in both the skin specimens and peripheral blood of patients with AD. In the current study, an increased proportion of Th17 and a decreased proportion of Tregs were observed in patients with AD, which is in accordance with the latter study. The possible explanation for this may be that transforming growth factor beta (TGF-β) is a major pluripotential cytokine with a pronounced immunosuppressive effect and the differentiation of Tregs from naive T-cell precursors needs the presence of TGF-β (31). Lower levels of TGF-β failure to effectively promote the development of Tregs, and insufficient TGF-β sensitivity makes it Th17 cells easy to escape the immune inhibition of Tregs, which leads to a decrease in Tregs and an increase in Th17 cells in AD (28). The present meta-analysis supports the causal role of the levels of Tregs as well as Th17 cells in the pathogenesis and development of AD.

In the present study, the pooled analysis of Th22 and Tregs showed high heterogeneity. The heterogeneity may primarily be from differences in disease severity. Furthermore, the subgroup analysis showed that patients with higher disease severity had higher levels of Th22 [mild: WMD = 1.33, 95% CI (1.24, 1.41), *p* < 0.001; moderate: WMD = 1.41, 95% CI (1.36, 1.54), *p* < 0.001; severe: WMD = 3.46, 95% CI (3.34, 2.81), *p* < 0.001] and lower levels of Tregs [mild: WMD = −1.43, 95% CI (−1.75, −1.11), *p* < 0.001; moderate: WMD = −2.16, 95%CI (−2.46, −1.86), *p* < 0.001; severe: WMD = −2.96, 95% CI (−3.25, −2.67), *p* < 0.001] in peripheral blood compared to healthy controls. No heterogeneity was observed. Therefore, the expression of Th22 and Tregs was associated with the severity of AD.

Our meta-analysis had several potential limitations. Firstly, only Chinese populations were included. Therefore, further studies need to be included to validate our results in a wider population in the future. Secondly, due to the limited data available in the original article, we were not able to perform a more comprehensive and detailed subgroup analysis, such as the correlation of the levels of the Th17 and IL-17 and patients with different severity of the disease. Thirdly, publication bias occurred in this study. Due to the initial search yielding eight relevant publications, the occurrence of selection bias in the results of the meta-analyses was therefore inevitable.

In conclusion, the present study found that patients with AD had an increased proportion of Th22 cells, Th17 cells, and IL-17, a decreased proportion of Tregs in peripheral blood, and the expression of Th22 and Tregs was associated with the severity of AD. These findings supported that the T helper and regulatory T cells might be involved in the pathogenic mechanisms of AD. Further studies with larger sample size and a broader population are needed to validate our results.

## Data Availability Statement

The original contributions presented in the study are included in the article/supplementary material, further inquiries can be directed to the corresponding author.

## Author Contributions

D-JZ and FH conceived the study, performed the literature search, and wrote the manuscript. TQ and H-XC analyzed and interpreted the data. D-JZ, FH, TQ and H-XC collected and assembled the data. H-XC submitted the manuscript. All authors read and approved the final manuscript.

## Conflict of Interest

The authors declare that the research was conducted in the absence of any commercial or financial relationships that could be construed as a potential conflict of interest.

## Publisher’s Note

All claims expressed in this article are solely those of the authors and do not necessarily represent those of their affiliated organizations, or those of the publisher, the editors and the reviewers. Any product that may be evaluated in this article, or claim that may be made by its manufacturer, is not guaranteed or endorsed by the publisher.
